# The Promotive Effects of Social Support for Parental Resilience in a Refugee Context: a Cross-Sectional Study with Syrian Mothers in Lebanon

**DOI:** 10.1007/s11121-019-0983-0

**Published:** 2019-01-25

**Authors:** Amanda Sim, Lucy Bowes, Frances Gardner

**Affiliations:** 1 Department of Social Policy & Intervention, University of Oxford, 32 Wellington Square, Oxford, OX1 2ER UK; 2 Department of Experimental Psychology, University of Oxford, Oxford, UK

**Keywords:** Social support, Refugees, Mental health, Parenting, Resilience

## Abstract

Exposure to war trauma increases the risk of negative individual and family-level outcomes. However, not all trauma-exposed individuals exhibit mental health or family dysfunction, and some function better than expected given their level of trauma exposure. Research with at-risk populations, including refugees, suggests that social support may promote positive mental health and parenting behavior in the face of adversity. We used cross-sectional data from 291 Syrian refugee mothers to test the role of perceived social support in promoting their psychological and parenting resilience, defined here as better than expected mental health and parenting behavior given level of exposure to war trauma. Psychological and parenting resilience were operationalized using the residual approach, which assesses the difference between mothers’ actual score on mental health and harsh discipline measures, and the score predicted by their level of trauma exposure. Linear regression models were used to test for associations between mothers’ perceived social support and their psychological and parenting resilience. Results showed that mothers’ perceived social support was associated with both psychological and parenting resilience, including after controlling for covariates. Exploratory analyses further suggested that emotional support, but not instrumental support, was associated with mothers’ psychological resilience. Social support may have a promotive effect on mothers’ mental health and parenting behavior in a context of ongoing conflict and displacement. Identifying intervention strategies to increase social support for refugees, within the framework of a multi-layered intervention approach, could potentially contribute to children’s psychosocial resilience via improved maternal mental health and parenting.

**Electronic supplementary material:**

The online version of this article (10.1007/s11121-019-0983-0) contains supplementary material, which is available to authorized users.

## Introduction

There is growing evidence that caregiver mental health and parenting are crucial to promoting the resilience of war-affected children (Reed et al. 2012; Tol et al. 2013). Yet, little is known about the variables and processes that promote the resilience of caregivers themselves. Resilience is usually defined as a “process encompassing positive adaptation within the context of significant adversity” (Luthar et al. 2000, p. 543). Drawing on this conceptualization, *parental resilience* can be defined as “the capacity of parents to deliver competent, quality parenting to children despite adverse personal, family, and social circumstances” (Gavidia-Payne et al. 2015, p. 111). While a preponderance of research focuses on the deleterious effects of war trauma on psychosocial functioning, resilience literature highlights the variation in how individuals respond to adverse circumstances, with some demonstrating extraordinary competence despite their level of risk (Masten 2001). Given the crucial role of caregivers in buffering children from the negative impacts of war, it is vital that resilience research moves beyond its traditional focus on the developmental trajectories of children to include exploration of the factors contributing to parental resilience.

Ecological approaches to understanding resilience posit that a range of variables at the individual, family, peer, and community levels interact with one another to increase or decrease the risk of negative outcomes in the face of adversity (Bronfenbrenner and Morris 1998). Among these variables, social support has received considerable attention in the theoretical and empirical literature. Social support has been defined as “social relationships that provide (or can potentially provide) material and interpersonal resources that are of value to the recipient, such as counseling, access to information and services, sharing of tasks and responsibilities, and skill acquisition” (Thompson et al. 2006, p. 2). The multidimensional nature of social support is reflected in the various approaches used to define and operationalize the construct. For example, types of support can include emotional and instrumental support, where emotional support involves providing care and empathy, and instrumental support involves providing assistance through giving of one’s time, skills, or other resources (Thompson et al. 2006).

Social support theory uses two models, the main effect and stress-buffering models, to conceptualize the association between social support and well-being. The main effect model posits that social support has a direct, beneficial effect on the outcome regardless of the level of stress exposure. The stress-buffering model, on the other hand, suggests that social support protects against negative effects only in the context of stressful experiences (Cohen and Hoberman 1983). In general, empirical studies have shown more consistent support for the direct, promotive effects of social support on well-being, with more inconclusive evidence on whether social support has buffering effects in times of stress (Rueger et al. 2016; Auerbach et al. 2011).

In this study, we examine the hypothesized promotive (i.e., main) effects of social support in the domains of mental health and parenting resilience among a highly vulnerable sample of Syrian refugee mothers. We chose to focus on social support as a promotive factor for psychosocial resilience because of the wealth of research on its beneficial effects among different at-risk populations, including those affected by armed conflict. For example, social support has been linked to fewer depressive symptoms among children and youth who were medically or psychiatrically ill, abused, or low-income (Rueger et al. 2016) and higher levels of prosocial behavior and adaptive functioning among former child soldiers in Sierra Leone (Betancourt et al. 2010). In a systematic review of 29 studies on the long-term mental health of refugees, lack of social support was associated with more depressive symptoms (Bogic et al. 2015).

Social support also has the potential to influence parenting behavior, both directly and indirectly. Belsky’s (1984) model of the determinants of parenting posits that social support shapes parenting through three main functions: providing emotional support, providing instrumental assistance, and setting or reinforcing social norms. For example, social support may influence parenting behavior through increased exposure to different parenting strategies. Previous research has found that mothers with larger social networks were more likely to praise their children and demonstrated less controlling behavior (Jennings et al. 1991). Studies have also found associations between social support and parenting behavior among parents experiencing adversity. A study with parents living in urban poverty found that more social support predicted greater increases in the frequency of positive parent-child interactions over time (Green et al. 2007). Less social support, on the other hand, increased the risk of child abuse among economically stressed mothers, as well as increased the effect of exposure to cumulative risk on child abuse potential (Ajduković et al. 2018). Several longitudinal studies concluded that greater maternal social support was associated with breaking the intergenerational cycle of abuse among mothers with a history of childhood maltreatment (Jaffee et al. 2013; Tracy et al. 2018). Social support may also promote positive parenting indirectly through improving the mental health of caregivers under stress, as evidenced by prior empirical work related to the Family Stress Model (Conger et al. 1992).

Finally, social support is of particular relevance in conflict settings as the degradation and destruction of support networks are a direct consequence of war. There have been some efforts to rebuild support structures as part of the response to the Syria refugee crisis, including peer-to-peer parenting support groups for refugee mothers in Lebanon (Mercy Corps n.d.), and a group-based psychosocial intervention for Syrian refugee and Jordanian youth with a focus on promoting social support (Panter-Brick et al. 2018). However, the paucity of studies on social support as a potentially modifiable target of intervention among war-affected caregivers warrants more in-depth investigation on its utility in promoting psychological and parenting resilience.

### The Present Study

In this study, we examine if social support is associated with psychological and parenting resilience among Syrian refugee mothers. Specifically, we test whether mothers’ perceived social support is associated with better than expected maternal mental health and parenting behavior given their level of past exposure to war-related trauma. Poor maternal mental health has been linked to negative parenting behavior in numerous studies: a meta-analysis of 46 studies, for example, showed that maternal depression was consistently associated with higher levels of harsh parenting (Lovejoy et al. 2000). Hence, this study examines the role of social support in promoting mothers’ psychological resilience as an important outcome in itself, as well as a significant correlate of parenting behavior.

We adopt the residual approach (Kim-Cohen et al. 2004) to operationalize psychological resilience as less emotional distress than predicted given mothers’ trauma exposure, and parenting resilience as less use of harsh punishment than predicted given mothers’ trauma exposure. Using cross-sectional data from a sample of 291 Syrian refugee mothers in Lebanon, we conducted regression analyses to determine if perceived social support was associated with mothers’ psychological and parenting resilience. We also conducted exploratory analyses on two specific dimensions of social support, emotional and instrumental support, to assess their relative contribution to mothers’ psychological and parenting resilience.

The study hypotheses were as follows:

1. Greater war trauma exposure would be associated with poorer maternal mental health, more rejecting parenting behavior, and greater use of harsh punishment.
2. Higher levels of perceived social support would be associated with greater psychological and parenting resilience (i.e., better than expected maternal mental health and parenting behavior given level of past war trauma exposure).

Our ultimate aim is to inform the development of psychosocial and parenting interventions by assessing whether social support—a potentially modifiable protective resource—contributes to more positive mental health and parenting outcomes among war-affected caregivers.

## Methods

### Procedure

Data for this study were collected from August 2016 to March 2017 as part of a baseline assessment for the International Rescue Committee’s (IRC) parenting program for Syrian refugees in Lebanon. The IRC is a non-governmental organization (NGO) providing economic, educational, and psychosocial services for Syrian refugees and vulnerable Lebanese communities. Sixteen female and two male IRC staff used community outreach meetings, home visits, and referrals to recruit Syrian caregivers of children aged 2 to 12 years (the target population for IRC’s parenting program) in Akkar, Arsal, Hermel, and Tripoli districts. Field staff explained the purpose and procedures of the study by reading aloud from a script and obtained verbal consent for participation. Given the vulnerable status of refugees, informed consent procedures emphasized that participation was voluntary and not linked to receiving assistance from the IRC or any other humanitarian organization.

All interviewers completed training on research ethics, informed consent, and interview skills prior to commencing survey administration using the application Open Data Kit on electronic tablets. Pictorial response ratings (e.g., circles of increasing size to depict Likert scales) were used during the interviews to aid participant comprehension. Individual interviews were conducted in Arabic at the participants’ home or the IRC office prior to participation in the parenting program. In response to adverse events such as participant distress or disclosure of abuse, interviewers followed a safety protocol that included procedures for referral to additional services if necessary. Interviewers also provided a list of local health and psychosocial support services if participants preferred. In order to respond to potential cases of child maltreatment, participant endorsement of the item “Beat him/her up, that is hit him/her over and over as hard as one could” were immediately flagged to the IRC child protection staff for follow-up. The study protocol was reviewed by senior IRC staff and approved by the University of Oxford Social Sciences and Humanities Inter-divisional Research Ethics Committee (Ref No: R46541/RE001).

### Measures

#### Sociodemographic Information

Sociodemographic data such as maternal age and education, length of displacement, and shelter type, were collected using items from the United Nations Vulnerability Assessment of Syrian Refugees in Lebanon (UNHCR et al. 2015).

### War Trauma Exposure

The Traumatic Events Checklist, adapted from the Harvard Trauma Questionnaire and previously used with Syrian refugee youth in Jordan, was used to assess mothers’ exposure to war trauma (Panter-Brick et al. 2018). The measure has 17 yes/no items such as “Been forcibly separated from your family” and “Been severely beaten.” Item scores were summed to obtain an overall measure of war trauma exposure.

### Maternal Mental Health

We assessed maternal mental health using the 21-item Depression Anxiety and Stress Scale (DASS) Short Form (Lovibond and Lovibond 1995). The Arabic language version of the original 42-item measure was previously validated with Arabic-speaking immigrants in Australia (Moussa et al. 2015). Items evaluated symptoms in the last 30 days such as “I couldn’t seem to experience any positive feeling at all” and “I felt I was close to panic.” Responses ranged from 0 (*Did not apply to me at all*) to 3 (*Applied to me very much, or most of the time*) (*α* = 0.91). The mean score in this sample falls above the cutoff score for “severe” depression, anxiety, and stress suggested by Lovibond and Lovibond (1995). However, there are no established norms for this study population as the measure has not been validated with Syrian refugees.

### Negative Parenting

The 24-item Parental Acceptance-Rejection Questionnaire (PARQ) Short Form was used to assess mothers’ rejecting parenting behavior (e.g., “I see my child as a big nuisance”) (Rohner and Khaleque 2008). The PARQ has been translated into 28 languages and used in more than 60 countries. The original PARQ consists of four subscales: warmth/affection, hostility/aggression, indifference/neglect, and undifferentiated rejection. In this study, positive items were reverse-coded and all items summed to achieve an overall measure of parental rejection. Item responses ranged from 0 (*Almost never true*) to 3 (*Almost always true*) in the last 30 days (*α* = 0.79).

We used eight items from the Discipline Module of UNICEF’s Multiple Indicator Cluster Survey (MICS) to assess mothers’ use of harsh punishment in the last 30 days (e.g., “Shouted, yelled, or screamed at him/her,” “Hit or slapped him/her on the face, head or ears”) (UNICEF 2016). The originally dichotomous items were modified in this study to a five-point Likert scale with responses ranging from 0 (*Never*) to 4 (*Almost all the time*) (*α* = 0.78).

### Social Support

The modified Medical Outcomes Study Social Support Survey (mMOS-SS) was used to assess mothers’ perceived social support (Sherbourne and Stewart 1991). The original 19 item-version covers four domains (emotional/informational support, tangible or instrumental support, positive social interaction, and affection). This study used the 8-item modified version that has two subscales for emotional (e.g., “How often is someone available to turn to for suggestions about how to deal with a personal problem?”) and instrumental (e.g., “How often is someone available to help you if you were sick in bed?”) support. Responses ranged from 0 (*None of the time*) to 4 (*All of the time)*. A validity study reported excellent psychometric properties of the 8-item mMOS-SS that were similar to those of the original measure (Moser et al. 2012). Cronbach’s coefficient alpha in this study was 0.88 for the eight items and 0.83 and 0.85 for the emotional and instrumental support subscales respectively.

### Covariates

We assessed relative socioeconomic disadvantage using a scale defined as follows: (1) residing in substandard housing including informal tented settlements, one-room structures, and converted or commercial spaces (e.g., factory, garage); (2) residing in overcrowded shelter, defined by the United Nations Humans Settlements Program as more than three people per room; (3) no working household member; and (4) experienced lack of food or money to buy food in the last 30 days. Summing across these four items provided a composite index of relative socioeconomic disadvantage, ranging from 0 to 4 (M = 2.89, SD = 1.11). To assess the validity of the index, we tested the correlation between the socioeconomic disadvantage score and a measure of daily stressors (Humanitarian Emergency Settings Perceived Needs Scale, World Health Organization and King’s College London 2011). The correlation coefficient was *r =* .481 (*p* < .001).

Other covariates included maternal education level, number of children, and sex and age of child. Covariates were selected based on prior research suggesting that these factors are associated with maternal well-being and parenting (Lansford et al. 2015; UNICEF 2010).

### Data Analysis

The residual approach to operationalizing resilience measures the continuum from vulnerability to resilience by using the standardized residual from a regression predicting the outcome from the risk exposure (Kim-Cohen et al. 2004). Further regression analyses are then conducted to determine if hypothesized protective factors are associated with resilience, as operationalized by residual scores. This approach, which has been used in several studies of resilience (Bowes et al. 2010; Collishaw et al. 2016), has the advantage of retaining the conceptualization of resilience as a continuum while avoiding the methodological challenges of detecting potentially small and unstable interaction effects (Luthar and Cushing 2002).

For this study, we first ran univariate linear regression analyses to test for associations between maternal war trauma exposure and maternal mental health, rejecting parenting behavior, and harsh punishment. Significant associations were retained for the next stage of analysis, in which mothers’ psychological and parenting resilience were defined by using standardized residuals from the respective regressions. Standardized residual scores were coded so that positive residuals indicate better than expected mental health and parenting behavior given level of war trauma exposure. For example, mothers whose actual mental health problem score was lower than their score as predicted by their level of war trauma exposure have a positive residual reflecting psychological resilience, while mothers whose actual mental health problem score was higher than their predicted score have a negative residual reflecting vulnerability. Finally, we conducted univariate linear regressions to test if mothers’ social support was associated with their psychological and parenting resilience. The proportion of missing data was less than 10% for all predictor, outcome, and control variables. Listwise deletion was used in all analyses.

We followed the STROBE (Strengthening The Reporting of OBservational Studies in Epidemiology) Checklist in reporting this study (please refer to the STROBE Checklist in Supplementary Information).

## Results

### Participants

A total of 292 caregivers were assessed for this study. As only one caregiver in the sample was male, data from this participant was not included in the analysis in order to clarify that the study pertains to mothers only (please refer to the Participant Flowchart in Supplementary Information). Participants’ sociodemographic information is summarized in Table 1. At the time of data collection, participants had been displaced in Lebanon an average of 2 to 3 years. The mean age of mothers was 31.83 years (*SD* = 8.18), and 53% had completed secondary school. The majority of participants were married (91.4%), 5.5% were widowed, and the remaining participants described themselves as in a relationship or separated/divorced. Of the married participants, 86.9% were cohabiting with their spouse at the time of the interview. On average, participants had three children and the mean age of the index child was 7.44 years (*SD* = 3.23). The majority of participants (69.1%) lived in substandard housing defined as informal tented settlements, one-room structures, and converted or commercial spaces, and 73% lived in overcrowded conditions (i.e., more than three persons residing in a room). Only 12% of participants had worked for income in the last 30 days, and almost half (47.1%) did not have a working member of the household. Participants reported lacking food or money to buy food an average of 13.73 days (*SD* = 7.87) days in the previous month. War-related trauma exposure averaged 7.21 events (*SD* = 3.48), with witnessing bombardment or explosion most commonly reported.

**Table 1:** Sample characteristics

	Mothers (*n* = 291) M (SD)	Minimum, maximum
Demographics		
Age (year)	31.83 (8.18)	19, 56
Marital status, married *n* (%)	266 (91.4)	–
Children under 18	3.4 (1.50)	1, 8
Age of index child	7.44 (3.23)	2, 12
Sex of index child, female *n* (%)	135 (46.4)	–
Believe in need for physical punishment, yes *n* (%)	47 (16.2)	–
War trauma (*N* lifetime events), 0–17	7.21 (3.48)	0, 15
Maternal mental health, 0–63	32.16 (13.18)	3, 62
Parental rejection, 0–72	20.78 (9.00)	1, 49
Harsh punishment, 0–32	9.47 (5.80)	0, 29
Social support, 0–32	15.43 (8.03)	0, 32

Data are mean (*SD*), unless otherwise indicated. Potential ranges are included next to each measure

### Is Mothers’ Exposure to War Trauma Associated with Maternal Mental Health and Parenting Behavior?

Mothers’ exposure to war trauma was associated with poorer maternal mental health (*β* = 0.42, *p* < 0.001) and greater use of harsh punishment (*β* = 0.13, *p* = 0.024), but not parental rejection (*β* = −0.01, *p* = 0.930). Parental rejection was thus dropped from subsequent analyses, and only the residual scores from regressing harsh punishment on war trauma exposure were used to represent parenting resilience.

### Maternal Psychological and Parenting Resilience

To derive measures of maternal psychological and parenting resilience, we saved and reverse-coded residual scores from regressing maternal mental health and harsh punishment on the level of war trauma exposure. Positive residual scores indicated mothers with better than expected mental health status given exposure to war trauma (i.e., psychological resilience, − 30.34 to 35.12) and mothers using less harsh punishment than expected given exposure to war trauma (i.e., parenting resilience, − 10.27 to 19.29). Scores for mothers’ psychological and parenting resilience were correlated (*r* = 0.24, *p* < 0.001).

### Associations between Covariates and Maternal Psychological and Parenting Resilience

Table 2 shows that higher maternal education level was associated with greater psychological and parenting resilience. Having more children was associated with less psychological resilience, but did not have a significant relationship with parenting resilience. The age of the index child was negatively correlated with mothers’ psychological resilience and positively correlated with parenting resilience, suggesting that mothers reporting on older children were less psychologically resilient but used less harsh punishment than predicted by their level of war trauma exposure. Child gender and relative socioeconomic disadvantage were not associated with either maternal psychological or parenting resilience.

**Table 2:** Associations between covariates and maternal psychological and parenting resilience

Covariates	Psychological resilience score	Parenting resilience score
	*β*	*β*
Relative socioeconomic disadvantage	− 0.03 [− 0.16, 0.09]	− 0.05 [−.17, 0.08]
Maternal education	0.22** [0.11, 0.34]	0.20** [0.09, 0.32]
Number of children under 18	− 0.20** [− 0.32, − 0.08]	− 0.07 [− 0.19, 0.50]
Age of index child	− 0.12* [− 0.24. 0.00]	0.13* [0.01, 0.24]
Sex of index child	.07 [− 0.05, 0.19]	.08 [− 0.04, 0.19]

Standardized regression coefficients, with 95% confidence intervals shown in brackets

Significance level shown as *p ≤ 0.05, **p ≤ 0.001

### Is Mothers’ Perceived Social Support Associated with Maternal Psychological and Parenting Resilience?

Univariate linear regressions indicated that mothers’ perceived social support was associated with greater psychological resilience (*β* = 0.24, *p* < 0.001) and parenting resilience (*β* = 0.18, *p* = 0.002). After controlling for relative socioeconomic disadvantage, maternal education, number of children, and age and sex of index child, the associations between mothers’ social support and their psychological (*β* = 0.23, *p* < 0.001) and parenting resilience (*β* = 0.18, *p* = 0.003) remained significant (Table 3).

**Table 3:** Linear regression models testing for associations between mothers’ perceived social support and psychological and parenting resilience to war trauma exposure

	Psychological resilience score		Parenting resilience score	
	Unadjusted	Adjusted	Unadjusted	Adjusted
Perceived social support	0.24** [0.12, 0.35]	0.23** [0.11, 0.36]	0.18* [0.07, 0.30]	0.18* [0.06, 0.29]

Standardized regression coefficients unadjusted and adjusted for covariates, with 95% confidence intervals shown in brackets. Covariates were socioeconomic disadvantage, maternal education, number of children, and age and sex of index child

Significance level shown as *p ≤ 0.05, **p ≤ 0.001

### Which Dimension of Social Support Is the Primary Driver of Maternal Psychological and Parenting Resilience?

We conducted secondary analyses using the emotional and instrumental subscales of the social support measure to assess whether a particular dimension of social support accounted for greater variance in mothers’ psychological and parenting resilience. The sum scores for the emotional and instrumental subscales were correlated (*r* = 0.58, *p* < .001). As each subscale contains only four items, this part of the analysis is primarily exploratory.

Multivariate linear regression analysis indicated that emotional support (*β* = 0.22, *p* = 0.004), but not instrumental support (*β* = 0.05, *p* = 0.655), was associated with greater psychological resilience. After controlling for covariates, the association between mothers’ emotional support and psychological resilience remained significant (*β* = 0.20, *p* = 0.008). While the overall measure of social support was associated with greater parenting resilience, the specific dimensions of emotional and instrumental support were not statistically significant (Table 4).

**Table 4:** Multivariate linear regression models testing for associations between emotional and instrumental support and psychological and parenting resilience to war trauma exposure

	Psychological resilience score		Parenting resilience score	
	Unadjusted	Adjusted for covariates	Unadjusted	Adjusted for covariates
Emotional support	0.22* [0.07, 0.37]	0.20* [0.05, 0.36]	0.12 [− 0.02, 0.26]	0.10 [− 0.05, 0.24]
Instrumental support	0.05 [− 0.10, 0.19]	0.06 [−0.10, 0.21]	0.09 [− 0.05, 0.23]	0.10 [− 0.05, 0.25]

Standardized regression coefficients unadjusted and adjusted for covariates, with 95% confidence intervals shown in brackets. Covariates were socioeconomic disadvantage, maternal education, number of children, and age and sex of index child

Significance level shown as *p ≤ 0.05, **p ≤ 0.001

## Discussion

The purpose of this study was to examine whether mothers’ perceived social support was associated with their psychological and parenting resilience in a context of ongoing war and displacement. Results were consistent with our hypothesis that social support would be associated with better than expected maternal mental health and parenting behavior given mothers’ past war trauma exposure. Furthermore, exploratory secondary analysis suggested that emotional support, rather than instrumental support, was the main driver of mothers’ psychological resilience. While further investigation is necessary given the limited number of measurement items, this finding is consistent with evidence that perceived emotional support protects against depression (Santini et al. 2015). In displacement settings characterized by high levels of need and scarce resources, it is likely that refugees are generally less able to provide instrumental support and more inhibited about seeking such support from their social network. For example, in a qualitative study with Syrian refugees in Lebanon, some parents pointed to the commonality of their difficult circumstances as a barrier to seeking and receiving help (Sim et al. 2018).

Surprisingly, regression analyses assessing the relationship between war trauma exposure and negative parenting found a significant association between war trauma and harsh punishment, but not rejection. The lack of association between war trauma and parental rejection runs counter to theoretical frameworks focusing on the negative impacts of traumatic events on parent-child communication, emotional responsiveness, and parents’ internal attachment representations of the self as caregiver (De Haene et al. 2010). However, there is little empirical evidence of the theorized link between parental war trauma exposure and rejecting parenting behavior as the majority of research has focused on child maltreatment rather than parental rejection as the outcome variable (Catani et al. 2008; Saile et al. 2014). Additional studies assessing different dimensions of negative parenting are necessary to determine if and why war trauma might have differential impacts on various forms of parenting behavior. For example, it is possible that the hyperarousal symptoms of post-traumatic stress, such as anger and irritability, are more directly associated with greater propensity for violence than with parental rejection, which encompasses a broader spectrum of negative parenting behaviors (e.g., indifference, neglect, lack of affection).

### Strengths and Limitations

This study contributes to the evidence base on the promotive effects of social support on mental health and parenting (Armstrong et al. 2005). While this evidence base is well-established in non-war settings, little is known about the role of social support in promoting psychological and parenting resilience among an adult refugee population. This study is unique in its examination of potential resilience processes among highly vulnerable refugee mothers living in a context of ongoing war and displacement. Theoretical and empirical work on resilience has focused almost exclusively on the outcomes of children, with very little known about how some parents are able to do well despite considerable odds (Luthar et al. 2006). This study seeks to address this gap in the literature by assessing the role of social support in promoting the resilience of mothers, thus bringing a family systems perspective to the study of resilience in conflict settings. Furthermore, it investigates resilience in different domains, reflecting the multidimensional conceptualization of the construct. Importantly, results showing the wide range of positive residual scores highlights the capacity for resilience even under conditions of extreme duress. This is an important finding in itself given the enduring emphasis in the literature on the psychological sequela of war trauma and serves as a reminder to investigate variables and processes that promote resilience in order to inform intervention development (Tol et al. 2013).

The study has several limitations. First, the sample, although recruited from four districts in Lebanon with a large refugee presence, was not representative. Thus, results cannot be generalized to the Syrian refugee population as a whole. Second, the cross-sectional nature of the data precludes causal inferences regarding the directionality of effects. Prior research has shown that depression and anxiety, for example, can lead to less support seeking, suggesting that the relationship between social support and mental health is transactional and bidirectional (Hammen 2006). Third, all measures were maternal self-report only, which increases the risk of bias associated with common rater effects (Podsakoff et al. 2003). It is possible that better mental health status contributed to more positive appraisal of social support, rather than the other way around. Fourth, while most of the measures used in this study have been validated with Arabic-speaking samples, they have not been validated with the Syrian refugee population. As such, it is not known whether the measures have adequate psychometric properties (e.g., test-retest reliability; criterion validity) when applied to this population or if there are culturally specific parenting practices or local idioms of distress that are not captured by these measures (Kirmayer 1989). Fifth, this study only examined two types of functional support, emotional and instrumental. Given the multidimensional nature of social support, future research should investigate additional dimensions of social support in relation to mental health and parenting among refugees, particularly the role of supportive intimate partner relationships given conceptual and empirical work positing its importance for positive parenting (Belsky 1984; Jaffee et al. 2013). Finally, the residual approach used in this paper to operationalize resilience rests on the assumption of a linear dose-response relationship between war trauma exposure and maternal mental health and parenting behavior. However, other possibilities exist, such as nonlinear threshold or inverted-U models (Masten and Narayan 2012). Furthermore, most war events checklists, including the measure used in this study, do not capture the recurring nature of war events, the timing of their occurrence, and the relative impact of specific events, all of which may alter the exposure-response relationship (Miller and Rasmussen 2014). The residual approach is an innovative and intuitive way of operationalizing resilience in terms of “doing better than expected” given level of risk exposure. We recognize, however, that resilience is a complex phenomenon grounded in culturally defined narratives and expectations of well-being (Panter-Brick 2015). Multiple and complementary approaches are therefore required to capture the normative as well as functional dimensions of resilience.

Some of these limitations relate to the constraints of conducting research in an ongoing refugee crisis. Longitudinal designs, which are more appropriate for inferring causality, are particularly challenging to implement in a volatile security context where participants may be difficult to track over time. Partnering with an NGO to recruit participants, while crucial for safe access and dissemination of findings, meant that the study sample was only composed of mothers since they comprise the vast majority of participants in the NGO’s parenting program. We were therefore unable to assess if social support operates differently for male and female caregivers, an important area for future research given evidence of the gendered nature of mental health and parenting (Panter-Brick et al. 2014).

## Conclusion

Study findings suggest that social support may have benefits for mothers’ psychosocial functioning. However, prior research calls for caution when targeting social support for intervention, particularly among highly stressed communities that face cultural and contextual barriers to accessing support. Qualitative research with Syrian refugee parents in Lebanon found that support-seeking was hampered by separation from extended family, mistrust of others in the community, and the perception that others were experiencing the same problems and thus unable to help (Sim et al. 2018). The same study also suggested that cultural norms may dissuade fathers, in particular, from seeking support from others outside the family. The daily stressors associated with displacement, and their effects on mental health, may also undermine caregivers’ motivation or efforts to seek support. Previous research has found that economically stressed individuals often feel overwhelmed and lack the time, energy, or hope to seek support from others (Thompson et al. 2006). In conditions of extreme adversity such as refugee settings, support-seeking may backfire if others are unable to provide support due to being in similarly difficult circumstances. There is some evidence in the literature of reverse stress-buffering effects, whereby the benefits of social support may be dampened under particularly stressful conditions (Auerbach et al. 2011). For example, a study with low-income, single mothers found that the positive influence of social support in promoting nurturing parenting and reducing punitive strategies diminished as neighborhood conditions worsened (Ceballo and McLoyd 2002). In sum, the same adverse conditions that contribute to social isolation may make the enlistment of social support especially challenging and weaken any potential positive effects.

Future research on the role of social support in developing resilience will need to consider the interaction between the individual and the unique ecological conditions engendered by forced displacement. In particular, research aimed at intervention development should incorporate a nuanced analysis of refugee caregivers’ environments, including the characteristics, capacities, and limitations of potential providers of support. Group-based parenting programs have shown some promise in addressing both the psychosocial and parenting needs of caregivers affected by war (Murphy et al. 2017). For example, a parenting intervention in northern Uganda had positive effects on maternal mental health and parenting practices, with mothers’ perceived social support mediating intervention effects on maternal depressive symptoms (Singla et al. 2015). Another parenting program for adolescents and their caregivers in South Africa had positive effects on caregivers’ social support (Cluver et al. 2018). It is possible that parenting interventions, which are often delivered in a group format, can be a vehicle through which caregivers increase their social networks, access emotional and informational support, and mutually reinforce positive behavior change. However, given the paucity of research on social support as a potential mechanism through which parenting interventions may improve caregiver mental health and parenting, more research on mediator and moderator effects, including potential reverse stress-buffering effects, is necessary. Prior research indicating the limitations of mutual help and support under certain adverse conditions suggests that simply improving social supports is not enough if those supports are under constant threat. Multi-layered interventions are necessary to promote refugees’ psychological and parenting resilience, from macro-level policy to address the social and structural determinants of psychosocial functioning, to targeted mental health and parenting interventions for those most at risk.

## Electronic supplementary material

ESM 1 (DOCX 32 kb)
