# Supplementary material for: The Promotive Effects of Social Support for Parental Resilience in a Refugee Context: a Cross-Sectional Study with Syrian Mothers in Lebanon
Source: Prev Sci. 2019 Jan 25;20(5):674–83. doi: 10.1007/s11121-019-0983-0 (PMC6541567; doi:10.1007/s11121-019-0983-0)
Supplement: Supplementary file 1 — (DOCX 32 kb) [file 11121_2019_983_MOESM1_ESM.docx]

**Participant Flowchart**

**Assessed for eligibility^a^**

N = 714

**Not eligible^b^**

N = 130

**Eligible**

N = 584

**Not selected**

N = 201

**Randomly selected to participate in study^c^**

N = 383

**Not interviewed^d^**

N = 91

**Interviewed**

N = 292

**Not included in analysis^e^**

N caregivers = 1

**Included in analysis**

N = 291

^a^ Eligibility criteria were: At least 18 years old; primary caregiver to a child aged between 2 and 12 years; originally from Syria.

^b^ No child in eligible age range (n = 120); did not consent (n = 10).

^c^ Caregivers were randomly selected by the lead author to be approached for interview due to insufficient time and human resources to assess all eligible caregivers.

^d^ Already participated in the parenting program (n = 14); could not locate/unavailable/declined (n=77)

^e^ As only one caregiver in the sample was male, data from this participant was not included in the analysis in order to make clear that the study pertains to mothers only.
